# Prognostic value of serum cystatin C and its changes in patients with ST-segment elevation myocardial infarction

**DOI:** 10.3389/fcvm.2026.1816496

**Published:** 2026-07-29

**Authors:** Nannan Wu, Guandong Li, Wenjie Ma, Mei Zhang

**Affiliations:** Department of Emergency, Changhai Hospital, The First Affiliated Hospital of Naval Medical University, Shanghai, China

**Keywords:** all-cause mortality, biomarker, cystatin C, dynamic change, prognosis, ST-segment elevation myocardial infarction

## Abstract

**Background:**

Serum cystatin C (Cys-C) has been established as a prognostic marker in various cardiovascular settings, but its changes and their relationship with long-term outcomes in ST-segment elevation myocardial infarction (STEMI) patients remain underexplored. Therefore, this study aims to evaluate the prognostic value of serum Cys-C concentration and its changes in predicting all-cause mortality in STEMI patients with normal or mildly impaired renal function.

**Methods:**

This single-center, retrospective observational study enrolled 753 STEMI patients who underwent emergency percutaneous coronary intervention between February 2019 and December 2024. Serum Cys-C levels were measured at baseline and the last measurement. Patients were categorized into tertiles based on baseline Cys-C, follow-up Cys-C, and the absolute change in Cys-C. The primary endpoint was all-cause mortality.

**Results:**

During follow-up of 627 days, 75 deaths occurred. Non-survivors had significantly higher baseline and follow-up Cys-C levels, as well as greater absolute increases in Cys-C, compared to survivors (all *p* < 0.001). Kaplan–Meier analysis showed significantly worse survival in the highest tertiles of baseline Cys-C (log-rank *p* < 0.001), follow-up Cys-C (*p* < 0.0001), and Cys-C change (*p* = 0.009). After multivariable adjustment, the highest tertile of baseline Cys-C was associated with a 2.20-fold increased risk of mortality (95% CI: 1.19–3.88, *p* = 0.012). The highest tertile of follow-up Cys-C was associated with a 3.12-fold increased risk of mortality (95% CI: 1.79–5.99, *p* < 0.001), while the highest tertile of Cys-C change conferred a 2.10-fold increased risk (95% CI: 1.14–3.68, *p* = 0.017).

**Conclusion:**

Elevated serum Cys-C levels at baseline and the last measurement, as well as an increase in Cys-C over time, are independently associated with higher all-cause mortality in STEMI patients. Serial measurement of Cys-C may enhance risk stratification and serve as a dynamic indicator for monitoring disease progression and treatment response.

## 1 Introduction

Cardiovascular diseases (CVDs) remain the leading cause of global mortality, accounting for approximately 31% of all deaths worldwide (1). In addition to their high fatality rate, CVDs—particularly myocardial infarction (MI)—are associated with substantial morbidity, placing a considerable economic burden on public health systems (2). MI is primarily caused by the occlusion of coronary arteries, leading to irreversible ischemic injury of cardiomyocytes due to inadequate oxygen and nutrient supply (3). As the world's most populous country, China is experiencing a rapidly growing burden of coronary atherosclerotic heart disease, the fundamental pathological basis of MI. It is projected that by 2030, there will be approximately 23 million new cases of acute myocardial infarction (AMI) annually in China (4). Among AMI subtypes, ST-segment elevation myocardial infarction (STEMI) represents the most critical condition due to its acute onset and high risk of adverse outcomes (5). Thus, the identification of reliable biomarkers for early risk stratification and prognosis in STEMI patients is of great clinical importance.

Cystatin C (Cys-C), a low-molecular-weight protein produced by all nucleated cells, functions not only as a potent inhibitor of cysteine proteases but also plays a role in intracellular protein metabolism (6), inflammation through neutrophil activation, and the regulation of extracellular matrix turnover (7). These properties suggest that Cys-C may be involved in the development and progression of atherosclerotic plaque (8).

Epidemiological studies have established Cys-C as a biomarker not only for renal function (9), but also for various cardiovascular conditions, including atherosclerosis, heart failure, ischemic stroke, and acute coronary syndromes (ACS) (10, 11). Prognostic value in non-ST-segment elevation ACS (NSTEMI) is well recognized for improving early risk stratification (12), and studies have also focused on the predictive role of preoperative Cys-C levels for outcomes in STEMI (13, 14). Elevated serum Cys-C at admission has been consistently associated with higher risks of mortality and fatal or non-fatal cardiovascular events in patients with both STEMI and NSTEMI (15). Specifically in STEMI, Cys-C emerges as a potential predictor for the no-reflow phenomenon after primary percutaneous coronary intervention (PPCI) (16). It also serves as an independent predictor of long-term mortality among STEMI patients undergoing late PCI (17). Moreover, Cys-C represents a novel biomarker with significant added prognostic value for STEMI patients treated with PPCI, improving the prediction of both short- and long-term clinical outcomes (18). Elevated admission levels of Cys-C have been independently associated with impaired myocardial perfusion, limited functional recovery, and the development of congestive heart failure (CHF) in anterior STEMI patients undergoing PPCI (19).

Despite these findings, there remains a scarcity of research examining the impact of preoperative Cys-C levels on all-cause mortality and their changes during follow-up in STEMI patients. Therefore, this study aims to evaluate the prognostic value of serum Cys-C concentration and its changes in predicting adverse cardiovascular outcomes in STEMI patients with normal or mildly impaired renal function.

## 2 Methods

### 2.1 Study design and population

This single-center, retrospective observational study aimed to evaluate the prognostic value of baseline serum Cys-C levels and their dynamic changes in patients with STEMI.

Between 15th February 2019 and 24th December 2024, a total of 1,594 patients with acute myocardial infarction undergoing emergency percutaneous coronary intervention (PCI) were assessed for eligibility. The study specifically included patients diagnosed with STEMI from whom biological samples were obtained upon arrival in the catheterization laboratory. Diagnostic criteria for STEMI comprised typical chest pain lasting >30 min accompanied by ST-segment elevation ≥2 mm in at least two contiguous electrocardiographic leads within 48 h of symptom onset (20). Exclusion criteria were: (1) age < 18 years; (2) history of cancer, concurrent autoimmune diseases, or mental disorders; (3) previous MI after percutaneous coronary intervention (PCI) or coronary artery bypass grafting (CABG); (4) estimated glomerular filtration rate (eGFR) < 60 mL/min/1.73 m^2^; and (5) significant valvular or structural heart disease.

The study protocol was approved by Shanghai Changhai Hospital Ethics Committee. All participants provided written informed consent. The study preserved participant anonymity and did not collect identifying information.

### 2.2 Data collection

Demographic and clinical data were systematically collected for all participants. The collected variables included age, gender, body mass index (BMI), and cardiovascular risk factors, including smoking status, hypertension, and diabetes. Smoking was defined as current active tobacco use. Hypertension was defined as systolic blood pressure ≥140 mmHg and/or diastolic blood pressure ≥90 mmHg measured on at least three occasions, or current use of antihypertensive medication. Diabetes was defined as the use of glucose-lowering agents or a fasting plasma glucose level ≥7.1 mmol/L.

Hemodynamic measures including systolic blood pressure (SBP), diastolic blood pressure (DBP), and heart rate were recorded at admission. Killip class was assessed to evaluate heart failure severity. The GRACE risk score was calculated for each patient to quantify ischemic risk. Door-to-balloon time was recorded as a key indicator of reperfusion timeliness. Information on medication use at discharge, including proton pump inhibitors (PPIs), angiotensin-converting enzyme inhibitors (ACEis)/angiotensin receptor blockers (ARBs), *β*-blockers, and mineralocorticoid receptor antagonists (MRAs), was collected from medical records. History of previous PCI was also collected.

Blood samples were collected at admission prior to coronary angiography using gel-containing vacutainer tubes, centrifuged within 1 h, and stored at −80 °C until analysis. Serum Cys-C was measured at baseline and the last measurement using enzyme-linked immunosorbent assay (R&D Systems kits). Reference intervals were 0.52–0.90 mg/L for women and 0.56–0.98 mg/L for men. Serum creatinine was used to estimate glomerular filtration rate (eGFR) via the MDRD formula. Fasting glucose, lipid profiles (TC, TG, HDL-C, LDL-C), BNP, NT-proBNP, and hsCRP were measured using an automated analyzer (HITACHI 7600, Japan).

### 2.3 Outcomes

Follow-up was conducted via telephone or clinical review for a minimum of 3 months to assess medication compliance and record adverse outcomes, including mortality verified through medical records or family interview. The primary endpoint was all-cause mortality, assessed over a follow-up period of up to 54 months (until 24th December 2024).

### 2.4 Statistical analysis

All analyses were performed using SPSS version 19.0. Normality of continuous variables was assessed using the Kolmogorov–Smirnov test and visual inspection of histograms and Q-Q plots. Continuous variables following a normal distribution are presented as mean ± standard deviation (SD), and group comparisons between survivors and non-survivors were performed using independent-sample t-tests. Continuous variables with a skewed distribution are presented as median with interquartile range (IQR), and group comparisons were performed using the Mann–Whitney U test. Categorical variables are expressed as numbers (percentages) and were compared using the chi-square test or Fisher's exact test, as appropriate.

Kaplan–Meier survival curves were plotted to visualize the association between serum Cys-C levels (including baseline, follow-up, and their difference) and all-cause mortality, and the log-rank test was used to compare survival differences across groups.

To further evaluate the association, baseline, follow-up serum Cys-C, and their difference, were categorized into tertiles, with the lowest tertile (T1) serving as the reference group. Cox proportional hazards models were used to estimate hazard ratios (HR) and 95% confidence intervals (CI) for the associations of these tertiles with mortality. Trends across tertiles were tested by entering the median value of each tertile as a continuous variable in the regression models. Three multivariate models were constructed: Model 1 was unadjusted; Model 2 was adjusted for age and gender; and Model 3 was further adjusted for age, gender, BMI, smoking, uric acid, TC, TG, Killip class, hypertension, diabetes, NT-proBNP, BNP, SYNTAX II score, GRACE risk score, previous PCI, use of PPIs, ACEis/ARBs, *β*-blockers, MRAs, and door-to-balloon time. Multicollinearity in Model 3 was assessed using variance inflation factor (VIF), with a VIF value > 5 indicating significant collinearity requiring further evaluation. In the present analysis, all covariates included in the final model demonstrated VIF values below the predetermined threshold, suggesting no substantial multicollinearity. Sensitivity analyses included additional adjustment for baseline Cys-C level and exclusion of patients who died within 6 months after admission. A two-sided *p*-value < 0.05 was considered statistically significant.

## 3 Results

### 3.1 Patient selection

**Figure 1:**
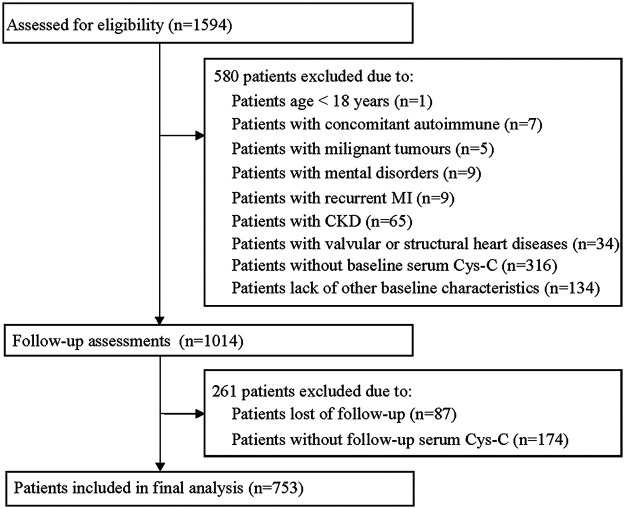
Flow chart of the patients selection.

As shown in Figure 1, after applying exclusion criteria: age < 18 years (*n* = 1), concomitant autoimmune diseases (*n* = 7), malignant tumours (*n* = 5), mental disorders (*n* = 9), recurrent MI (*n* = 9), chronic kidney disease (CKD) with eGFR < 60 mL/min (*n* = 65), significant valvular or structural heart disease (*n* = 34), absence of baseline serum Cys-C measurement (*n* = 316), and lack of other baseline characteristics (*n* = 134), 1,014 patients entered follow-up. Among these, 261 were further excluded due to loss to follow-up (*n* = 87) or missing follow-up Cys-C data (*n* = 174), resulting in 753 patients included in the final analysis.

### 3.2 Patient characteristics

The overall mean age was 63.63 ± 12.61 years, and the majority were men (71.0%, *n* = 535). During a median follow-up period of 627 days (IQR: 452–1,035 days), a total of 75 deaths from all causes occurred. The median interval between the baseline and follow-up Cys-C measurements was 8 days (IQR: 6–12 days) for the overall cohort.

Comparative analysis between survivors and non-survivors revealed significant differences in multiple baseline characteristics (Table 1). Patients who died were older, had a higher body mass index, and exhibited a higher prevalence of smoking. They also presented with elevated systolic and diastolic blood pressures, as well as higher levels of fasting plasma glucose, total cholesterol, and triglycerides, along with a longer door-to-balloon time. Furthermore, the non-survivor group had significantly worse cardiac function as assessed by Killip class, higher rates of hypertension and diabetes, and markedly elevated levels of cardiac biomarkers and risk scores. In terms of medication use at discharge, a higher proportion of non-survivors received proton pump inhibitors (PPIs) compared to survivors (69.3% vs. 56.5%, *p* = 0.044), while the use of ACEis/ARBs, *β*-blockers, and MRAs did not differ significantly between the two groups (all *p* > 0.05).

**Table 1:** Baseline characteristics of the included patients.

Variable	Overall (*n* = 753)	Survival (*n* = 678)	Death (*n* = 75)	P
Age, years	63.63 ± 12.61	63.21 ± 12.63	67.42 ± 11.76	0.006
Gender, n(%)				
Men	535 (71.0)	477 (70.4)	58 (77.3)	0.258
Women	218 (29.0)	201 (29.6)	17 (22.7)	
BMI, kg/m^2^	23.99 ± 3.70	23.84 ± 3.72	25.31 ± 3.21	0.001
Smoking, n(%)				
No	496 (65.9)	456 (67.3)	40 (53.3)	0.022
Yes	257 (34.1)	222 (32.7)	35 (46.7)	
SBP, mmHg	118.19 ± 12.19	117.72 ± 12.18	122.38 ± 11.54	0.002
DBP, mmHg	73.91 ± 6.76	73.61 ± 6.75	76.66 ± 6.32	<0.001
Heart rate, beats/min	75.62 ± 4.33	75.57 ± 4.26	76.15 ± 4.95	0.272
Uric acid, mg/dL	335.63 ± 20.59	335.15 ± 20.86	339.92 ± 17.46	0.057
FPG, mmol/L	5.31 ± 0.32	5.30 ± 0.32	5.42 ± 0.29	0.003
TC, mmol/L	4.83 ± 0.35	4.82 ± 0.35	4.92 ± 0.33	0.015
TG, mmol/L	1.20 ± 0.11	1.20 ± 0.11	1.24 ± 0.11	0.003
Killip class, n(%)				
I	538 (71.4)	517 (76.3)	21 (28.0)	<0.001
II	124 (16.5)	102 (15.0)	22 (29.3)	
III	62 (8.2)	47 (6.9)	15 (20.0)	
IV	29 (3.9)	12 (1.8)	17 (22.7)	
Hypertension, n(%)				
No	309 (41.0)	287 (42.3)	22 (29.3)	0.041
Yes	444 (59.0)	391 (57.7)	53 (70.7)	
Diabetes, n(%)				
No	527 (70.0)	483 (71.2)	44 (58.7)	0.034
Yes	226 (30.0)	195 (28.8)	31 (41.3)	
NT-proBNP, pg/mL	1,589 (1,152, 2,001)	1,425 (1,028, 1,936)	1,856 (1,326, 2,214)	<0.001
BNP, pg/mL	375 (286, 524)	364 (263, 507)	401 (286, 554)	<0.001
SYNTAX II score	40.78 ± 5.08	40.41 ± 5.00	44.09 ± 4.61	<0.001
GRACE risk score	138.61 ± 16.53	137.39 ± 16.25	149.66 ± 14.90	<0.001
Previous PCI, n(%)				0.561
No	700 (93.0)	632 (93.2)	68 (90.7)	
Yes	53 (7.0)	46 (6.8)	7 (9.3)	
**Medication use**				
PPI, n(%)				0.044
No	318 (42.2)	295 (43.5)	23 (30.7)	
Yes	435 (57.8)	383 (56.5)	52 (69.3)	
ACEi or ARB, n(%)				0.644
No	182 (24.2)	166 (24.5)	16 (21.3)	
Yes	571 (75.8)	512 (75.5)	59 (78.7)	
*β*-blocker, n(%)				0.247
No	165 (21.9)	153 (22.6)	12 (16.0)	
Yes	588 (78.1)	525 (77.4)	63 (84.0)	
MRA, n(%)				0.141
No	632 (83.9)	574 (84.7)	58 (77.3)	
Yes	121 (16.1)	104 (15.3)	17 (22.7)	
Door-to-balloon time, min	148 (67, 578)	147 (68, 588)	157 (72, 579)	<0.001
Length of stay (days)	11 (8, 14)	12 (8, 14)	10 (7, 13)	0.177
Length of repeated measurements of Cys-C (days)	8 (6, 12)	9 (6, 13)	7 (6, 13)	0.105
Follow-up duration, days	366 (162, 638)	345 (159, 666)	388 (172, 691)	0.301
Serum Cys-C, mg/L	1.23 ± 0.3	1.21 ± 0.37	1.45 ± 0.34	<0.001
Serum Cys-C follow-up, mg/L	1.01 ± 0.16	1.07 ± 0.15	1.14 ± 0.15	<0.001
Serum Cys-C difference, mg/L	0.219 (0.009, 0.512)	0.204 (0.008, 0.488)	0.294 (0.010, 0.551)	<0.001

All values are expressed as mean ± standard deviation, median [interquartile range (IQR)], or number (%).

BMI, body mass index; SBP, systolic blood pressure; DBP, diastolic blood pressure; FPG, fasting plasma glucose; TC, total cholesterol; TG, triglyceride; BNP, brain natriuretic peptide; cystatin C, Cys-C; PPI, proton pump inhibitor; ACEi, angiotensin-converting enzyme inhibitor; ARB, angiotensin receptor; MRA, mineralocorticoid receptor antagonist.

Notably, serum Cys-C levels were significantly higher among non-survivors both at baseline (1.45 ± 0.34 vs. 1.21 ± 0.37 mg/L, *p* < 0.001) and at follow-up (1.14 ± 0.15 vs. 1.07 ± 0.15 mg/L, *p* < 0.001). The absolute change in Cys-C (difference) was also greater in those who died [0.294 (0.010–0.551) vs. 0.204 (0.008–0.488) mg/L, *p* < 0.001].

### 3.3 Prognostic value of serum Cys-C and its changes

**Figure 2:**
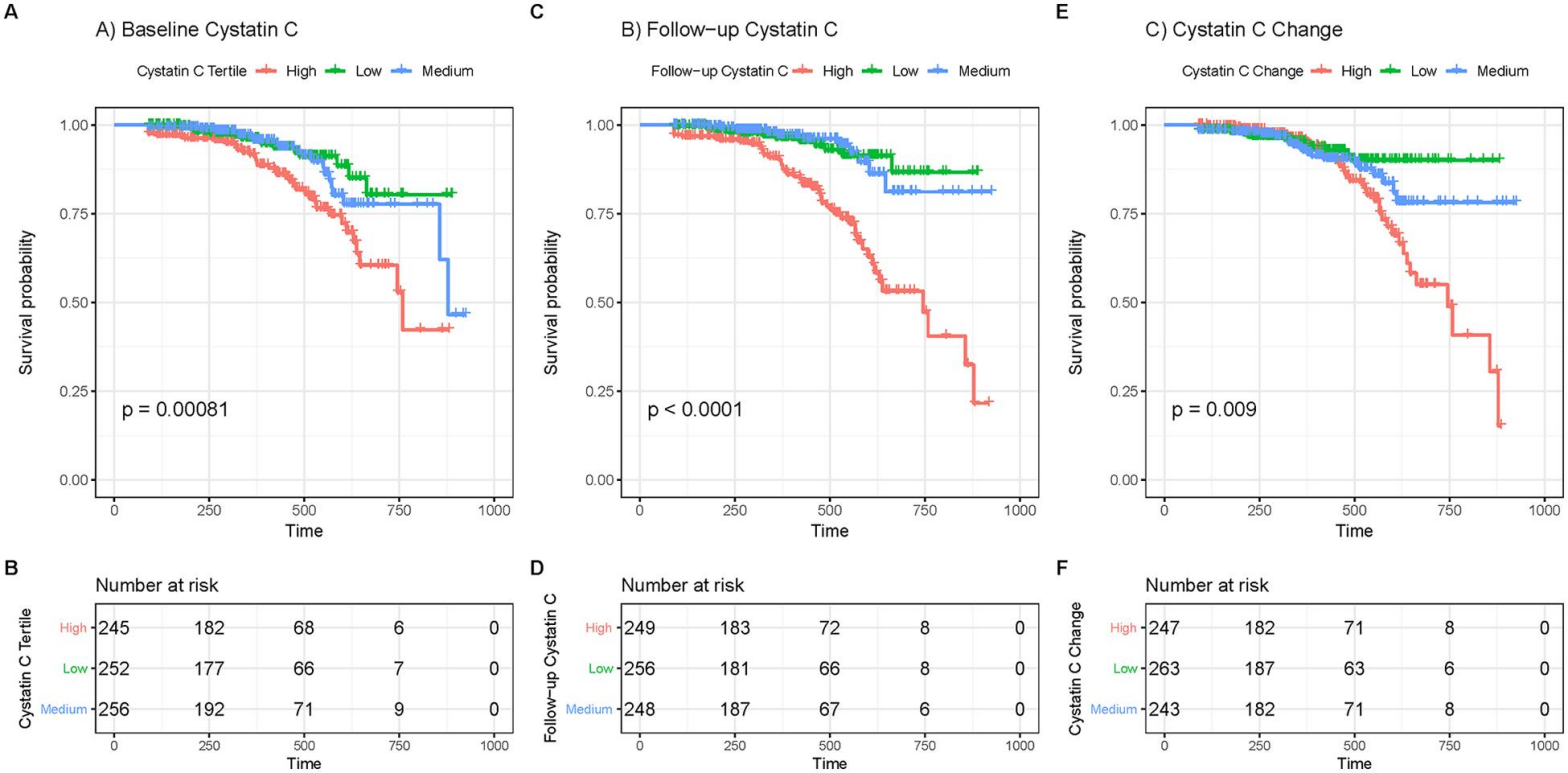
Kaplan–meier curves for overall survival of patients with ST-segment elevation myocardial infarction on serum Cys-C stratification results. **(A)** baseline serum Cys-C; **(B)** follow-up serum Cys-C; and **(C)** serum Cys-C change.

Kaplan–Meier survival curves for all-cause mortality according to tertiles of serum Cys-C are shown in Figure 2. Log-rank tests revealed significant differences in survival probability among the tertile groups for all three Cys-C measures. Patients in the highest tertile (T3) of baseline serum Cys-C (Figure 2A) had significantly worse survival compared to those in the lower tertiles (Log-rank *p* < 0.001). Similarly, a more pronounced separation of curves was observed for follow-up Cys-C levels (Figure 2B), with the highest tertile associated with the poorest survival outcome (Log-rank *p* < 0.0001). Furthermore, the magnitude of Cys-C change from baseline to follow-up also significantly stratified patient survival (Figure 2C), as a greater increase in Cys-C (highest tertile of change) was associated with a markedly higher mortality rate (Log-rank *p* = 0.009).

The associations of serum Cys-C levels (baseline, follow-up, and their difference) with all-cause mortality are presented in Table 2. Using the lowest tertile (T1) as the reference, Cox regression analyses revealed a significant increase in mortality risk associated with the highest tertile (T3) of baseline serum Cys-C. In the fully adjusted model (Model 3), the hazard ratio (HR) for T3 was 2.20 (95% CI: 1.19–3.88, *p* = 0.012), with a significant positive trend across tertiles (*p* for trend=0.006).

**Table 2:** HRs and 95% CIs of overall mortality according to tertiles of serum Cys-C.

Variables	Model 1		Model 2		Model 3	
	HR (95%CI)	P	HR (95%CI)	P	HR (95%CI)	P
Serum Cys-C, mg/L						
T1	Ref.		Ref.		Ref.	
T2	1.31 (0.68–2.55)	0.420	1.25 (0.64–2.44)	0.509	1.13 (0.57–2.20)	0.711
T3	2.68 (1.48–4.87)	0.001	2.60 (1.43–4.72)	0.002	2.20 (1.19–3.88)	0.012
*P* for trend	<0.001		0.001		0.006	
Serum Cys-C follow-up, mg/L						
T1	Ref.		Ref.		Ref.	
T2	0.98 (0.43–2.23)	0.968	0.99 (0.44–2.25)	0.987	0.89 (0.39–2.01)	0.745
T3	4.32 (2.31–8.10)	<0.001	4.27 (2.28–8.01)	<0.001	3.12 (1.79–5.99)	<0.001
*P* for trend	<0.001		<0.001		<0.001	
Serum Cys-C difference, mg/L						
T1	Ref.		Ref.		Ref.	
T2	1.40 (0.72–2.70)	0.319	1.43 (0.74–2.76)	0.287	1.31 (0.67–2.45)	0.399
T3	2.35 (1.29–4.27)	0.005	2.37 (1.30–4.31)	0.005	2.10 (1.14–3.68)	0.017
*P* for trend	0.003		0.004		0.014	

Model 1: unadjusted factor.

Model 2: adjusted for age and gender.

Model 3: adjusted for age, gender, BMI, smoking, uric acid, TC, TG, Killip class, hypertension, diabetes, NT-proBNP, BNP, SYNTAX II score, and GRACE risk score, previous PCI, use of PPI, ACEi or ARB, *β*-blocker and MRA, and door-to-balloon time.

A more pronounced risk was observed for the highest tertile of follow-up Cys-C. After full adjustment, patients in T3 had a 3.12-fold increased risk of mortality (95% CI: 1.79–5.99, *p* < 0.001) compared to those in T1. A strong positive dose-response relationship was evident (*p* for trend < 0.001). Similarly, a greater increase in Cys-C from baseline to follow-up (highest tertile of Cys-C difference) was independently associated with higher mortality. The fully adjusted HR for T3 was 2.10 (95% CI: 1.14–3.68, *p* = 0.017), and the trend across increasing tertiles remained statistically significant (*p* for trend=0.014).

### 3.4 Sensitivity analyses

Sensitivity analyses were performed to assess the robustness of the primary findings (Supplementary Table 1). Two approaches were employed: (1) exclusion of patients who died within the first 6 months after admission, and (2) additional adjustment for baseline serum Cys-C level in the models for follow-up Cys-C and Cys-C difference. Overall, the magnitude of the associations was attenuated in these sensitivity analyses; however, most associations remained statistically significant. Notably, the association between the highest tertile of serum Cys-C difference and mortality borderlined on statistical significance after excluding early deaths (*P* for trend = 0.065).

## 4 Discussion

This retrospective observational study demonstrates that elevated serum Cys-C levels, both at baseline and at follow-up, as well as the change in Cys-C, are independently associated with an increased risk of all-cause mortality in STEMI patients. These associations persisted after extensive adjustment for established cardiovascular risk factors, cardiac biomarkers, and risk scores.

Our findings are consistent with and extend previous research on the prognostic value of Cys-C specifically in STEMI. Prior studies have firmly established that a single elevated Cys-C level at admission is a powerful predictor of adverse outcomes in this population (21, 22). Silva et al. (18) demonstrated that baseline Cys-C was a strong predictor of both in-hospital progression to cardiogenic shock and long-term mortality in STEMI patients treated with primary PCI, independent of left ventricular function. Similarly, Chen et al. (17) showed that high admission Cys-C levels predicted long-term mortality in STEMI patients undergoing late percutaneous coronary intervention. Furthermore, research by Cheng et al. (16) and Tang et al. (19) identified Cys-C as a significant predictor for the no-reflow phenomenon and impaired myocardial perfusion and functional recovery, respectively, highlighting its role in microvascular dysfunction and poor recovery post-STEMI. While these studies focused on a single baseline measurement, our study provides significant novel insight by highlighting the critical importance of monitoring the dynamic change in Cys-C levels over time. Our analysis demonstrates that patients with persistently high or increasing Cys-C levels follow-up after the event constitute a particularly vulnerable subgroup, facing a markedly elevated risk of death (fully adjusted HR: 3.12 for follow-up Cys-C).

Elevated cystatin C (Cys-C) is increasingly recognized as a significant predictor of adverse outcomes in STEMI patients (23), operating through interconnected mechanisms including endothelial dysfunction, oxidative stress, and inflammatory activation (24). As a sensitive marker of renal function and a cysteine protease inhibitor, Cys-C contributes to microvascular impairment and poor clinical prognosis (25). It promotes endothelial dysfunction by reducing nitric oxide bioavailability and increasing adhesion molecule expression, leading to microvascular obstruction and the no-reflow phenomenon post-PPCI (26, 27). Concurrently, Cys-C is associated with heightened oxidative stress via reactive oxygen species generation and impaired antioxidant defense, exacerbating myocardial injury (28). Furthermore, Cys-C correlates with elevated inflammatory markers such as CRP and IL-6, fostering a pro-inflammatory state that aggravates plaque instability and impedes tissue repair (29). Notably, a significant decrease in Cys-C levels further suggests a protective effect, potentially indicating successful reperfusion and reduced inflammatory burden, which correlates with improved outcomes.

Sensitivity analyses, which involved excluding patients who died within the first 6 months and further adjusting for baseline cystatin C levels, yielded attenuated yet largely consistent results. Although the association between the magnitude of cystatin C change and all-cause mortality lost formal statistical significance after excluding early deaths (*p* for trend = 0.065), the persistence of a positive trend and elevated hazard ratios suggests that the observed relationship is unlikely to be entirely due to reverse causality. The reduction in statistical significance in this specific analysis is likely attributable to the diminished sample size and statistical power rather than a true absence of association, underscoring the robustness of the primary findings while highlighting the need for larger studies to confirm the prognostic importance of dynamic changes in cystatin C.

The association between long-term outcome and changes in Cys-C over time were first studied in our study, which puts forward a potential target for the management of STEMI patients after surgery. Immunoturbidimetry were the most commonly used methods for the laboratory dosage of Cys-C and have good precision and specificity. Therefore, the use of these methods by most of the studies brings greater reliability to our results.

Several limitations should be considered in the interpretation of our findings. First, as a single-center retrospective study with a modest sample size of 753 patients, the generalizability of our results may be limited. The potential for selection bias inherent in non-randomized designs must also be acknowledged. Future studies involving larger, multi-center cohorts are needed to validate our observations. Second, despite extensive statistical adjustment for known confounders, residual confounding from unmeasured or unaccounted factors cannot be excluded. Third, the observational nature of this study precludes the establishment of causal relationships between serum cystatin C levels and clinical outcomes. Additionally, the lack of data on secondary cardiovascular events limits our ability to assess the impact of cystatin C or its dynamic changes on recurrent events, although cardiovascular deaths are presumed to constitute a major portion of all-cause mortality in this cohort. Moreover, the absence of established cutoff values or reference ranges for cystatin C dynamics hinders precise sensitivity analyses and mortality prediction. Finally, the inclusion of only Chinese patients may affect the extrapolation of our findings to other ethnic populations. Further research incorporating diverse populations and exploring the integration of cystatin C with established risk stratification tools, such as the GRACE score, may enhance predictive accuracy for long-term outcomes in STEMI patients (30). Furthermore, although the interval between Cys-C measurements was comparable between groups and unlikely to have confounded our primary findings, the absolute change in Cys-C does not account for variations in observation time across individuals. Future studies incorporating the rate of change (i.e., *Δ*Cys-C per unit time) may provide a more refined assessment of Cys-C dynamics and further improve prognostic accuracy.

## 5 Conclusions

In this retrospective observational study, we found that elevated serum Cys-C levels at baseline and a significant increase in Cys-C during follow-up are all independently associated with reduced long-term survival in STEMI patients. These findings underscore the value of serum Cys-C not only as a robust prognostic biomarker for identifying high-risk individuals following an acute coronary event but also as a dynamic indicator for monitoring disease progression and treatment response.

## Data Availability

The raw data supporting the conclusions of this article will be made available by the authors, without undue reservation.
