# Supplementary material for: Prognostic value of serum cystatin C and its changes in patients with ST-segment elevation myocardial infarction
Source: Front Cardiovasc Med. 2026 Jul 29;13:1816496. doi: 10.3389/fcvm.2026.1816496 (PMC13461404; doi:10.3389/fcvm.2026.1816496)
Supplement: Supplementary file 1 [file Table1.docx]

Supplemental table 1. Sensitivity analyses of HRs and 95% CIs of overall mortality according to tertiles of serum Cys-C

|  | Excluding death in previous 6 months | |  | Adjustment for baseline serum cystatin C | |
| --- | --- | --- | --- | --- | --- |
|  | HR (95%CI) | P |  | HR (95%CI) | P |
| Serum Cys-C, mg/L |  |  |  |  |  |
| T1 | Ref. |  |  | - | - |
| T2 | 1.04 (0.43-2.56) | 0.883 |  | - | - |
| T3 | 2.11 (1.02-3.15) | 0.046 |  | - | - |
| **P for trend** | **0.039** |  |  | **-** |  |
| Serum Cys-C follow-up, mg/L |  |  |  |  |  |
| T1 | Ref. |  |  | Ref. |  |
| T2 | 0.87 (0.30-1.99) | 0.801 |  | 0.80 (0.35-1.99) | 0.681 |
| T3 | 3.01 (1.60-5.58) | <0.001 |  | 2.56 (1.16-4.38) | 0.002 |
| **P for trend** | **<0.001** |  |  | **0.002** |  |
| Serum Cys-C difference, mg/L |  |  |  |  |  |
| T1 | Ref. |  |  | Ref. |  |
| T2 | 1.15 (0.54-2.22) | 0.801 |  | 1.22 (0.64-2.511) | 0.466 |
| T3 | 1.89 (0.93-3.38) | 0.064 |  | 1.99 (1.03-3.80) | 0.046 |
| **P for trend** | **0.065** |  |  | **0.048** |  |

Adjusted for age, gender, BMI, smoking, uric acid, TC, TG, Killip class, hypertension, diabetes, NT-proBNP, BNP, SYNTAX II score, and GRACE risk score, pprevious PCI, use of PPI, ACEi or ARB, β-blocker and MRA, and door-to-balloon time.
